# Concussion Incidence and Recurrence in Professional Australian Football Match-Play: A 14-Year Analysis

**DOI:** 10.1155/2017/2831751

**Published:** 2017-07-19

**Authors:** Nathan Gibbs, Mark Watsford

**Affiliations:** ^1^South Sydney Sports Medicine, 111 Anzac Pde, Kensington, Sydney, NSW 2033, Australia; ^2^Sydney Swans Football Club Pty Ltd., Driver Ave, Moore Park, Sydney, NSW 2021, Australia; ^3^Faculty of Health, University of Technology Sydney, P.O. Box 123, Broadway, Sydney, NSW 2007, Australia

## Abstract

**Background:**

Concussion incidence rates in professional Australian football may be underreported due to the injury classification definition. A myriad of factors contribute to concussion risk; however, there is limited long-term surveillance in Australian football. This study analysed concussion in one Australian football team over an extended period.

**Method:**

Match-play concussion injuries in one team (*n* = 116 participants) were diagnosed and treated by the team physician over 14 years. Analysis of factors related to concussion including matches played, time of day and season, and return to play provided an insight into occurrence and recurrence rates.

**Results:**

140 concussions were recorded (17.6 per 1000 player match hours). A strong relationship was evident between matches played and concussion incidence (*r* = 0.70) and match conditions did not negatively affect the concussion rate. Whether an athlete returned to play in the same match or suffered a loss-of-consciousness concussion (*p* = 0.84), their ensuing rate of concussion was not affected.

**Conclusion:**

Concussion in professional Australian football was related to the number of matches played. Further, neither previous incidence nor loss of consciousness affected future concussion risk. This study provides ecologically valid evidence of the concussion incidence rate in professional Australian football and has implications for the management of athletes sustaining concussion injuries.

## 1. Introduction

Australian football is the major professional national football code played in Australia. Substantial amounts of running, accelerating, decelerating, and skill execution are required for success [1–3]. Match-play also involves contests for ball possession and repetitive contact between players involving tackling and wrestling. Injuries are common and when considering the premier competition, the Australian Football League (AFL), 38–46 new injuries per club have been recently reported per year [4]. Such incidence renders up to 18% of the playing list at any club unavailable at any given time during a season due to injury [4]. These injuries range from muscle strains to compound fractures and include concussion.

Concussion injury is common in contact and collision sports and may affect vision, postural stability, memory, behaviour, and mood [5]. Professional sports where frequent collisions lead to concussion incidence, such as rugby league and American football (NFL), have reported rates of 28.3 and 18.1 concussions per 1000 player match hours (pmh), respectively [6, 7]. An analysis of concussion in the AFL between 2000 and 2003 revealed an incidence rate of 5.6 per 1000 pmh [8]. Whilst concussion injury occurs in AFL, it may be underreported in surveillance studies due to underreporting by players [9] and the classification methods used for injury. Since surveillance studies typically classify injuries when the player subsequently misses at least one match [4], concussion may not be accurately included as players tend not to miss the following week's match. The most recent report on injuries in the AFL, via a concussion audit, noted that concussion incidence was 6.0 per 1000 player hours (95% confidence interval: 4.39–7.65) when considering all concussion injuries, whether players missed a match or not [4]. More recently, match-play concussion injuries are diagnosed as players unable to pass the sport concussion assessment tool test within 20 minutes, therefore potentially omitting some concussions.

Along with the contact nature of the game, some circumstantial factors may contribute to concussion risk. There is an absence of research examining the role of environmental conditions on concussion. The speed of match-play, resultant impact velocity, or alterations in ground traction may be affected by whether the match is held during the day or night or in dry or wet conditions. Despite a long-term commitment to injury surveillance, true concussion rates in the AFL remain unknown. Given the uncertainty surrounding concussion rates in professional sport, along with the potential long-term implications from concussion injury and the elevated risk of recurrent concussion as it relates to exposure time [10], the primary objective of this study was to provide an accurate synopsis of incidence in the AFL. Further, the relationship of concussion incidence to number of matches played, weather conditions, match time, and risk of recurrence was examined, along with the rate of loss-of-consciousness (LOC) concussions and return-to-play outcomes.

## 2. Materials and Methods

This study analysed match-play concussion injuries in one professional AFL team over 14 years (2000–2013). The lead author was the sports physician who attended all matches during this period and was responsible for diagnosis, management, and return-to-play decisions. Factors related to concussion incidence were examined with a view to providing insight into occurrence and recurrence rates in AFL.

One AFL team was analysed with senior-level players (*n* = 116) from all in-season and finals matches over the 14 years included in the analysis. The sample represented a successful AFL team, with 11 finals appearances and two premierships during the period. The procedures of the study were approved by the Human Research Ethics Committee at the University of Technology Sydney. The participants provided informed consent to use their injury data and be involved in the study and their rights were protected.

Concussion was defined as the immediate and transient posttraumatic impairment of neural function and was typically caused by any force to the head (direct or indirect). Symptoms and signs of concussion included LOC, appearing dazed or unsteady, disorientation, memory loss, blurred vision, slowed response rate, irritability, altered personality, headache, or reduced coordination and balance [5, 11, 12]. These measures were used exclusively in the clinical diagnosis as they have been described as the primary foci for the determination of concussion incidence. All incidents of these indicators were recorded as cases of concussion, regardless of the severity, as these symptoms reflect neurological impairment. There is debate about including concussion injuries which do not include unconsciousness or amnesia; however, each case was included in the current study [13] resulting in a highly sensitive dataset. Some of these cases are underreported elsewhere due to a range of injury definitions.

The acute clinical features of concussion typically resolved spontaneously. The physician made an immediate diagnosis upon witnessing the injury or a replay and assessing signs and symptoms in the injured player. Further, the physician used a series of standard verbal questions about any symptoms the player experienced during the previous quarter of the match, including reference to typical concussion symptoms such as blurred vision, seeing stars, slowed thinking, headache, confusion, memory loss, feeling off balance, and nausea. Following concussion, some players were interchanged and assessed off the field, whilst less obviously concussed players were assessed during match breaks. The latter typically reported experiencing concussion symptoms in the previous quarter which had resolved.

As soon as was practically possible, any loss of consciousness, unsteadiness, or amnesia was resolved. Subsequently, signs of concussion such as orientation, lucidity, balance, speed of response, concentration, short- and long-term memory, personality changes, and coordination were assessed. Postconcussion symptoms, for example, headache or blurred vision, were also recorded. Following resolution of these symptoms, players were required to perform an exercise test (short sprints, reaction skill drills with the football) to assess coordination and any return of symptoms. If symptoms did not return and the player appeared clinically normal, they were permitted to return to the same game. This procedure was revised for the final year of this study (2013), with the AFL mandating that players with diagnosed concussion were not permitted to return to play on the day of injury.

Following the match, all concussed players followed the same treatment protocol regardless of whether they continued playing in the game, returned to play in the same game, or did not continue to play following the onset of their concussion. All concussed players were reviewed 24–48 hours after game by the physician and rested for at least 72 hours, with all activity being below symptomatic thresholds. Provided that they were symptom-free and clinically normal upon examination, players then commenced low-intensity/low-vibration exercises, for example, stationary cycling and low-load resistance training. If they remained symptom-free, training included low-velocity running the following day and they returned to normal training approximately five days after concussion, avoiding body-contact drills. In the absence of posttraining symptoms, players were cleared to play in the next game, usually seven days after the concussion was sustained. Neuropsychometric testing was not routinely performed on the players during the study period; rather, coordination was tested with sport-specific drills, for example, short sprints and football skill drills, to assess handling, kicking, and reaction time.

The concussion incidence rate in AFL match-play and the number of recurrences per player were calculated at the end of the 14-year period. Recurrent concussion was defined as any subsequent concussion injury that occurred after a player had returned to full team participation from the index concussion [14]. The data was presented in two ways: the incidence rate was calculated as the number of concussions per 1000 pmh, using the convention that each match involved 18 players on the field at one time for a total of 1.33 hours. This method has been used previously to determine incidence rate [7]. Alternatively, to scrutinise match exposure and the cohort of players who sustained concussion, the number of matches per concussion was calculated; this data was not related to match duration; rather, it was based on there being 22 players per team, per match. This value was calculated as the total number of matches played by each individual divided by the number of concussions sustained by the individual and was limited to players who had sustained at least one concussion (due to the inability to use a zero score on the denominator). The influence of time of season was considered (regular season or finals), and environmental conditions were assessed at each match with matches classed as “dry” or “wet” (a subjective inference of the prevailing conditions throughout the match) and “day” or “night” (earlier/later than 7:00 pm start time) fixtures.

Given the reported relationship between concussion incidence and acute reconcussion risk [10, 15, 16], incidence data was also analysed solely for the players who returned to play in the same match. Since all players who returned to play in the same match missed some match-time due to medical assessment, the remaining available match-time was calculated in terms of the number of quarters remaining in the match. This was subsequently transformed into a value representing the number of matches (by dividing by 4) to align with the other indices. Finally, concussions involving LOC, defined as being motionless on the ground with eyes closed and not responding to verbal stimuli, were noted. The use of the Glasgow coma scale was considered for periods of unconsciousness greater than one minute.

The analysis was delimited to senior football matches per player at the club during the 14-year period. Matches for other clubs were not considered, nor were concussion injuries sustained during training, due to the inability of the chief physician to analyse the concussion immediately and the poor precision of player recall for prior concussion injuries [9].

Descriptive statistics were calculated for concussion incidence rates, the relationship with environmental conditions, and return-to-play characteristics. Parametric statistical tests were used as the central-limit theorem ensures that the sampling distribution is close enough to normal for accurate inferences, even when sample sizes are small [17]. The relationship between the number of concussions and number of matches played was calculated using Pearson's product-moment correlations with magnitude interpreted according to Hopkins [17]. To analyse concussions relative to number of matches played, a one-way analysis of variance with Scheffe post hoc tests was calculated for the players who sustained at least one concussion (players with zero instances were not considered). Players were categorised by number of matches played during the study period (1–50, 51–100, 101–150, 151–200, and >201 matches). Finally, to compare the effect of a LOC concussion on recurrence, an independent *t*-test was performed on the matches per concussion index to compare players sustaining LOC and non-LOC concussions. An alpha level of *p* < 0.05 was used for all statistical procedures.

## 3. Results

There were 333 matches during the study period (7972 pmh). Concussion injuries were recorded from 45 different players, representing 38.8% of players.  Table 1 provides descriptive information for incidence and other factors that were investigated. When considering match exposure, concussion occurred every 52.3 matches per player, which equates to one concussion injury every 2.28 seasons per player.  Table 1 also presents the descriptive environmental data pertaining to time of match, weather conditions, and whether the match was a regular season or finals fixture. No statistical tests were performed for these variables.

**Figure 1:**
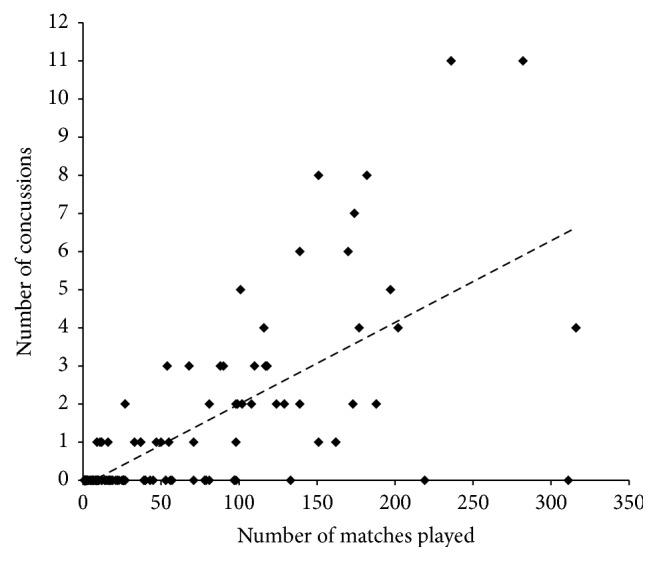
Relationship between concussion incidence and matches played. Equation of regression line: *y* = 0.0214*x* − 0.15; *r*^2^ = 0.484.

**Figure 2:**
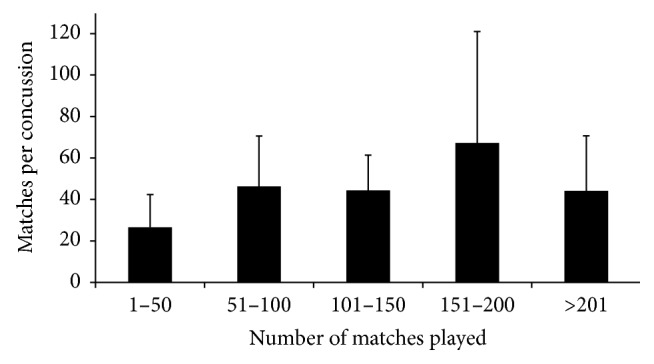
Matches per concussion injury for players who sustained at least one concussion, grouped by number of matches played (values represent mean ± SD).

As depicted in Figure 1, Pearson's correlation analysis revealed a “very large” relationship between the number of matches played and number of concussion injuries (*r* = 0.70). For players who sustained at least one concussion, Figure 2 reveals no differences in recurrent incidence rate considering the number of matches played (*F* = 2.16; *p* = 0.09).  Table 1 displays the descriptive data for players who sustained concussion but returned to play in the same match. One player sustained a recurrent concussion in the same match after medical clearance to return to play. Accordingly, the recurrence rate following return to play in the same match using the accumulated time from the quarters of match-play was one concussion every 52.75 matches.

Fifteen players sustained 17 LOC concussion injuries, accounting for 12% of all concussions (Table 1). For the cohort of players who sustained concussion, there was no difference in incidence rate between those sustaining an LOC concussion and those who did not lose consciousness (47.3 ± 38.6 versus 45.2 ± 29.9 matches per concussion, resp.; *p* = 0.84).

No postconcussion ongoing symptoms were reported. No concussed player missed the subsequent week's match due to their concussion. Considering the subsequent week's matches for each concussion, one player sustained a recurrent concussion (5.37 (95% CI: 0.14–29.93) per 1000 pmh). Furthermore, no player retired from Australian football because of an inability to recover from postconcussion symptoms or recurrent concussions.

## 4. Discussion

Concussion is an inherent risk of all contact sports. This study longitudinally examined one AFL team to gauge the incidence and recurrence of concussion, with a concussion injury rate of 17.6 per 1000 pmh reported. It is the first study to objectively report concussion information from one AFL club and provides numerous salient findings for medical staff from numerous sports. Concussion incidence was strongly related to the number of matches played, increasing by approximately one concussion for every 50 AFL matches played. The “very large” correlation between the number of concussions in a player's career and the number of AFL matches played (Figure 1) revealed that 48% of the variance in concussion incidence was derived from the number of matches played and indicates that match exposure is a primary contributor to concussion risk. The remaining 52% of unexplained variance in incidence is likely related to other physical parameters, skill components, or other factors.

The current results of 17.6 concussions per 1000 pmh with 12% involving LOC and 80.7% returning to play on the same day provide objective data across a broad timeframe, indicating a similar incidence to other contact sports. When compared to other contact sports, an incidence rate of 18.1 per 1000 pmh has been computed in the NFL [6] and a longitudinal study in rugby league revealed an incidence of 28.3 per 1000 pmh [7]. Three of the last four years in the latter study revealed a significantly increased incidence of >30 per 1000 pmh [7]. Considering professional soccer, it has been speculated that there was a 50% and 22% probability of males and females, respectively, sustaining a concussion over a 10-year playing career [18].

Concussion injury may be underreported in the AFL injury survey over the last decade (~0.4–1.0 incidences per club per season) [4]. This is primarily due to the definition of injury involving one missed match, which rarely occurs after a concussion. The reported AFL concussion rate was 5.6 per 1000 pmh [8]; however, this may be underreported due to interreliability issues accompanying multiple data collection modalities, including the retrospective nature of data collection potentially missing some cases [19]. Furthermore, less significant concussion injuries where the player passed cognitive/coordination testing and continued playing were likely not recorded as concussion injuries. Since match-play stoppage is not immediate for injury in AFL, many players who sustain quickly resolving concussion symptoms likely remain undetected. Accordingly, the use of the more inclusive concussion definition in this study enables the most robust analysis of concussion incidence to date. Even the recently noted incidence rate of 6.0 concussions per 1000 player hours [4] appears to underreport the true rate based on the findings of the current study. The methodology of the “concussion audit” reported in the recent AFL injury report [4] is unclear; however, the current results provide a robust, long-term observation of concussion incidence from the same medical professional in the same team environment.

Despite the fact that there are no statistical tests incorporated, wet weather conditions revealed a tendency to reduce the risk of concussion from 18.8 to 10.4 per 1000 pmh when compared with matches in dry conditions (Table 1). This may be due to slower movement velocities generated by players on softer ground surfaces or more cautious match-play in an attempt to protect ball possession or due to a loss of traction resulting in slipping immediately prior to the point of impact which may reduce collision forces. Whether the game was played at night or during the daytime did not seem to affect the concussion rate (Table 1). Appropriate lighting and adequate specific preparation may negate the proprioceptive challenges faced by athletes performing at nighttime.

Returning to play on the same day was not associated with a higher rate of subsequent concussion in this cohort. 80.7% of players returned to play in the same match and the recurrent concussion rate from these players (one per 52.75 matches, Table 1) was almost identical to the normal incidence rate (one per 52.3 matches), indicating that returning to play on the same day does not lead to an elevated concussion risk. Changes to concussion laws in the AFL may lead to alterations in future return-to-play statistics. Accordingly, it is important to monitor the level of reporting and underreporting of symptoms by players under these new laws to maintain player well-being and effective surveillance procedures.

No players missed the following week's game due to their concussion. One player in the current study recorded a subsequent concussion in the following week's match. This reinjury rate of 5.37 concussions per 1000 pmh tended to be lower than the normal concussion rate. When correctly managed, acute concussion appears to resolve promptly and does not adversely affect subsequent performance [8] or player selection/availability or increase the rate of further concussion. Furthermore, longer recovery does not reportedly influence return-to-play performance [20].

Research investigating ice hockey [21], rugby [16], American football [15], Canadian football [10], and soccer [22] reveals a 3–6 times increased rate of concussion in athletes with a prior history of concussion. However, some of these studies are constrained by patient recall and inaccuracies associated with omitting immediate diagnosis. Interestingly, one study using patient recall to assess concussion history reported that 31% of 486 retrospectively diagnosed concussions had not been diagnosed at the time they occurred [9]. Further, other authors have reported deficits in neurocognitive capacity following football match-play where no concussion was diagnosed [23]. Such inconsistencies were likely eliminated in the current study using one expert physician to acutely diagnose concussion.

Recurrent concussion rate was not related to prior concussion or a longer playing career as there was no elevated risk for any group of players when they are categorised by career length (Figure 2). Interestingly, the 151–200 matches' group tended to display a reduced concussion rate and the 1–50 matches' group tended to demonstrate an increased risk. The tendency in the 1–50 matches' group could be related to their short playing career or relative inexperience, potentially due to poor physical and skill capacities or perhaps different playing positions. An examination of the physical and skill attributes related to concussion should be considered in future research.

Returning to play on the same day following medical clearance from acute concussion in AFL footballers was not related to the recurrence rate. Further, career length did not appear to be related to recurrent concussion risk; however, the long-term effects of repeated concussions remain unclear. The consensus statement from the 4th International Conference on Concussion in Sport concluded that a cause and effect relationship was yet to be demonstrated between chronic traumatic encephalopathy and concussions or exposure to contact sports [5]. Further research is required to definitively determine any causal relationships with concussion risk.

The incidence rate of LOC concussion was low (12% of cases), with all concussions resolving spontaneously within 60 seconds. The brief period of LOC sometimes associated with concussion does not necessarily reflect severity or predict recovery time [24]. Further, whilst LOC may indicate serious intracranial injury, symptoms in all concussions in the current study were transient, did not deteriorate, and did not require radiological investigation. LOC concussions reportedly contribute to an increased rate of recurrent concussions [10] and previous concussions may enhance the rate of future LOC concussions [9]; however, the current results revealed that LOC concussion was not related to a subsequent elevation in incidence. No difference was evident between concussion rates for the 15 players who sustained a LOC concussion and the 30 players who sustained non-LOC concussions (47.3 versus 45.2 matches per concussion, resp.). Further research should investigate this issue to ascertain a risk profile for recurrent concussion risk.

The longitudinal examination of one AFL team provides a dataset with high ecological validity. One limitation of this study was that players' concussion history prior to the 14-year study period was not recorded, nor was any follow-up possible once they departed the team. Furthermore, training or lower level match-play concussions were not included in the dataset; however, these omissions ultimately strengthened the research design and avoided errors associated with player recall, ensuring an accurate dataset for analysing match-play concussions. The implications from these findings are therefore limited to AFL-level match-play and should not be extrapolated to other tiers of Australian football. In addition, the sole use of a symptom checklist in the clinical diagnosis process, as opposed to cognitive or eye-tracking assessments, creates some uncertainty around the true concussion incidence rate in AF. The use of other clinical diagnosis strategies may indeed yield higher (or lower) incidence rates. Despite this uncertainty, the diagnosis being performed by the same physician for the entire duration of the study ensures the reliability of the measure and contributes positively to the impact of the study. Finally, the level of self-reported concussion symptoms also remains a limitation in all concussion research, including the current study; however the use of one sole physician to diagnose and treat all concussion injuries is likely to have improved symptom reporting by the players due to heightened familiarity and trust.

## 5. Conclusion

There are limitations in assessing concussion in Australian football due to traditional injury definitions; therefore, true incidence rates remain unknown. This research objectively reported concussion incidence for one AFL club over 14 years. A relatively high concussion rate was evident compared to previous research, incidence was strongly related to the number of matches played, and previous concussion was not associated with increased rate of future concussions. Environmental conditions did not appear to negatively affect the incidence rate. Returning to play in the same match once medically cleared did not increase the reconcussion risk and sustaining a LOC concussion was not related to higher subsequent concussion incidence rates. Given the monitoring strategy utilised which relied upon diagnosis and management with one single physician over the course of the study, the results provide ecologically valid evidence of the concussion incidence rate in professional Australian football and have implications for concussion management.

## Figures and Tables

**Table 1:** Descriptive results for concussion incidence considering time of season, environmental conditions, loss of consciousness, and return to play (*n* = 116 players).

Condition (number of games)	Number of concussions	Incidence rate per 1000 pmh (95% CI range)
Total (333)	140	17.6 (14.77–20.72)

Regular season (308)	130	17.6 (14.73–20.94)
Finals (25)	10	16.7 (8.01–30.70)

Day (183)	73	16.6 (13.03–20.90)
Night (150)	67	18.6 (14.42–23.64)

Wet (52)	13	10.4 (5.55–17.81)
Dry (281)	127	18.8 (15.70–22.41)

Loss of consciousness concussion	17	2.1 (1.24–3.41)
Non-loss of consciousness concussion	123	15.4 (12.82–18.41)

Did not return to play in same game	27	
Cleared and returned to play in same game	113	
(i) Returned to play for 1 quarter	48	
(ii) Returned to play for 2 quarters	32	
(iii) Returned to play for 3 quarters	33	

*Note*. pmh: player match hours.
